# Host-biting rate and susceptibility of some suspected vectors to *Leishmania braziliensis*

**DOI:** 10.1186/1756-3305-7-139

**Published:** 2014-03-31

**Authors:** Morgana Michele Cavalcanti de Souza Leal Diniz, Fredy Galvis Ovallos, Claudia Maria de Castro Gomes, Cecilia de Oliveira Lavitschka, Eunice Aparecida Bianchi Galati

**Affiliations:** 1 Postgraduate Program in Public Health, School of Public Health, University of São Paulo – USP, São Paulo, SP Brazil; 2 Department of Pathology, University of São Paulo Medical School, São Paulo, SP Brazil; 3 Professional Improvement Program, PAP-FUNDAP-Department of Epidemiology/School of Public Health, University of São Paulo- USP, São Paulo, Brazil; 4 Department of Epidemiology, School of Public Health, University of São Paulo – USP, São Paulo, SP Brazil

**Keywords:** Phlebotominae, Host biting rate, Vectorial capacity, *Leishmania (Viannia) braziliensis*, Vector

## Abstract

**Background:**

American tegumentary leishmaniasis is a serious Brazilian public health problem. This diseases is attributed to seven species of *Leishmania*, however, the majority of cases are associated with *Leishmania braziliensis*. Some phlebotomine species have been implicated in the transmission of this parasite, nonetheless only *Psychodopygus wellcomei* has had its vectorial competence demonstrated. Thus this study sought to assess some parameters related to the vectorial capacity of anthropophilic species of sand fly occurring in São Paulo state: *Pintomyia fischeri, Migonemyia migonei Nyssomyia intermedia, Nyssomyia whitmani, Expapillata firmatoi* and *Psychodopygus ayrozai,* under laboratory conditions. These parameters were the duration of the gonotrophic cycle, proportion of females which feed on hamster, the rate of infection by *L. braziliensis* and the duration of the extrinsic incubation period.

**Methods:**

The sandflies were collected in three regions of the São Paulo state: Greater São Paulo and the Mogi Guaçu and Iporanga municipalities. To assess the proportion of engorged females the insects were fed on hamsters to estimate the duration of the gonotrophic cycle. To estimate the susceptibility to infection of each species, their females were fed on hamsters infected with *Leishmania braziliensis* and dissected to ascertain the localization of the flagellates and estimate the extrinsic incubation period.

**Results:**

Low hamster attractiveness to *Ps. ayrozai* was observed. A high proportion of engorged females was observed when the hamster had its whole body exposed. The gonotrophic cycle ranged between three and eight days. *Mg. migonei, Pi. fischeri, Ny. neivai, Ny. intermedia, Ny. whitmani* and *Ex.firmatoi* presented susceptibility to infection by *L. braziliensis*. The highest infection rate (34.4%) was observed for *Ny. whitmani* and the lowest for *Ny. intermedia* (6.6%). *Mg. migonei* presented late-stage infection forms on the fifth day after feeding, but in the other species these forms were observed as from the fourth day.

**Conclusions:**

Our results, together with other parameters of their behavior under natural conditions, suggest the potential role of *Ex. firmatoi* as vector of this parasite and reinforce that of *Mg. migonei, Pi. fischeri, Ny. neivai, Ny. intermedia* and *Ny. whitmani* in the areas in which they occur.

## Background

American tegumentary leishmaniasis (ATL) may be characterized as a complex of diseases caused by 14 species of the *Leishmania* genus that infect humans, some affecting the skin and others the skin and the mucous membranes [1-3]. It is a zoonosis involving a variety of reservoirs represented by wild animals, synanthropic and domestic animals, and vectors [4,5]. Its importance for public health is due to the damage it causes to the tissues, the psychosocial impact on the people affected, the disability adjusted life years lost (DALYs) in the populations [5] and its wide geographical distribution in the Central and South America, apart from Chile and Uruguay which have no recorded cases [6,7].

This disease has been attributed in Brazil to seven species of *Leishmania: Leishmania (L.) amazonensis), L. (Viannia) braziliensis, L. (V.) guyanensis, L. (V.) lainsoni, L. (V.) shawi, L. (V.) naiffi* and *L. (V.) lindenbergi*. The majority of cases are associated with *L. braziliensis* which presents the most widespread geographical distribution and is associated with cases of the mucocutaneous form. The wild rodents *Oryzomys concolor*, *O. capito*, *O. nigripes*, *Akodon arviculoides*, *Proechyms* sp. *Rattus rattus* and *Rhipidomys leucodactylus* and the opossum *Didelphis marsupialis* have been found as natural hosts of this parasite [8].

Although various phlebotomine species have been implicated in the transmission of *L. braziliensis*[2,8], only *Psychodopygus wellcomei* has had its vectorial competence demonstrated [9]. However, on the basis of such criteria as anthropophily, high frequencies in foci of ATL, the finding of natural infections by flagellates of this parasite or when the parasites are identified as *Leishmania* (*Viannia*) sp. in areas where the human cases have been attributed to it, other phlebotomine species have been implicated in the transmission.

In ATL endemic areas in the state of São Paulo, the sand fly species *Nyssomyia intermedia*, *Nyssomyia neivai*, *Nyssomyia whitmani*, *Migonemyia migonei*, *Pintomyia fischeri* and *Pintomyia pessoai* have been indicated as possible vectors of *L. (V.) braziliensis*[10,11]. *Pintomyia fischeri* and *Mg. migonei* are species widely distributed in São Paulo state and are noteworthy as they are highly anthropophilic, presenting considerable density in woodland and peridomiciliary environments [12,13].

Beyond that, *Pi. fischeri* has been indicated as a possible vector of the ATL agent [12] due to its susceptibility to experimental infection [14] and in the state of Espírito Santo, its natural infection by *L. (V.) braziliensis* has been demonstrated [15].

*Expapillata firmatoi* is an anthropophilic species which bites humans in both the diurnal and nocturnal periods and is one the most frequent species in the northeastern region of São Paulo state [16]. It has been suspected of being a vector of *Leishmania (Leishmania) infantum* in foci of visceral leishmaniasis in Rio de Janeiro [17] and its presence although in low densities is frequently cited in research undertaken in these two states and also in Paraná.

*Psychodopygus ayrozai* is a sandfly frequently collected in the Atlantic biome and is highly anthropophilic [18-20]. In Amazonian region it has been implicated as a vector of *Leishmania naiffi*[21,22].

Beyond its vectorial competence, the vectorial capacity of a hematophagous insect involves other parameters, such as density of the insect population in relation to the host, the biting habit (measured by the proportion of females which feed on a particular host divided by the duration of the gonotrophic cycle), the survival rate, the period of extrinsic incubation of the parasite and the proportion of the infected insects which became infective [23-25].

The obtaining of such parameters contributes to the assessment of the potential of phlebotomine populations in the transmission of ATL agents, the basis for the formulation of measures of prevention and control of this disease. Thus, this study sought to assess some parameters of anthropophilic species of sand fly occurring in São Paulo state: *Pi. fischeri, Mg. migonei Ny. intermedia, Ny. whitmani, Ex. firmatoi* and *Ps. ayrozai* under laboratory conditions for investigate their potential as vectors of *L. braziliensis*. These parameters comprise the proportion of females which feed on a susceptible host (hamster), duration of the gonotrophic cycle, the rate of infection by this parasite; the proportion of females with late-stage infection forms among those infected by this parasite and the duration of the extrinsic incubation period.

## Methods

**Figure 1:**
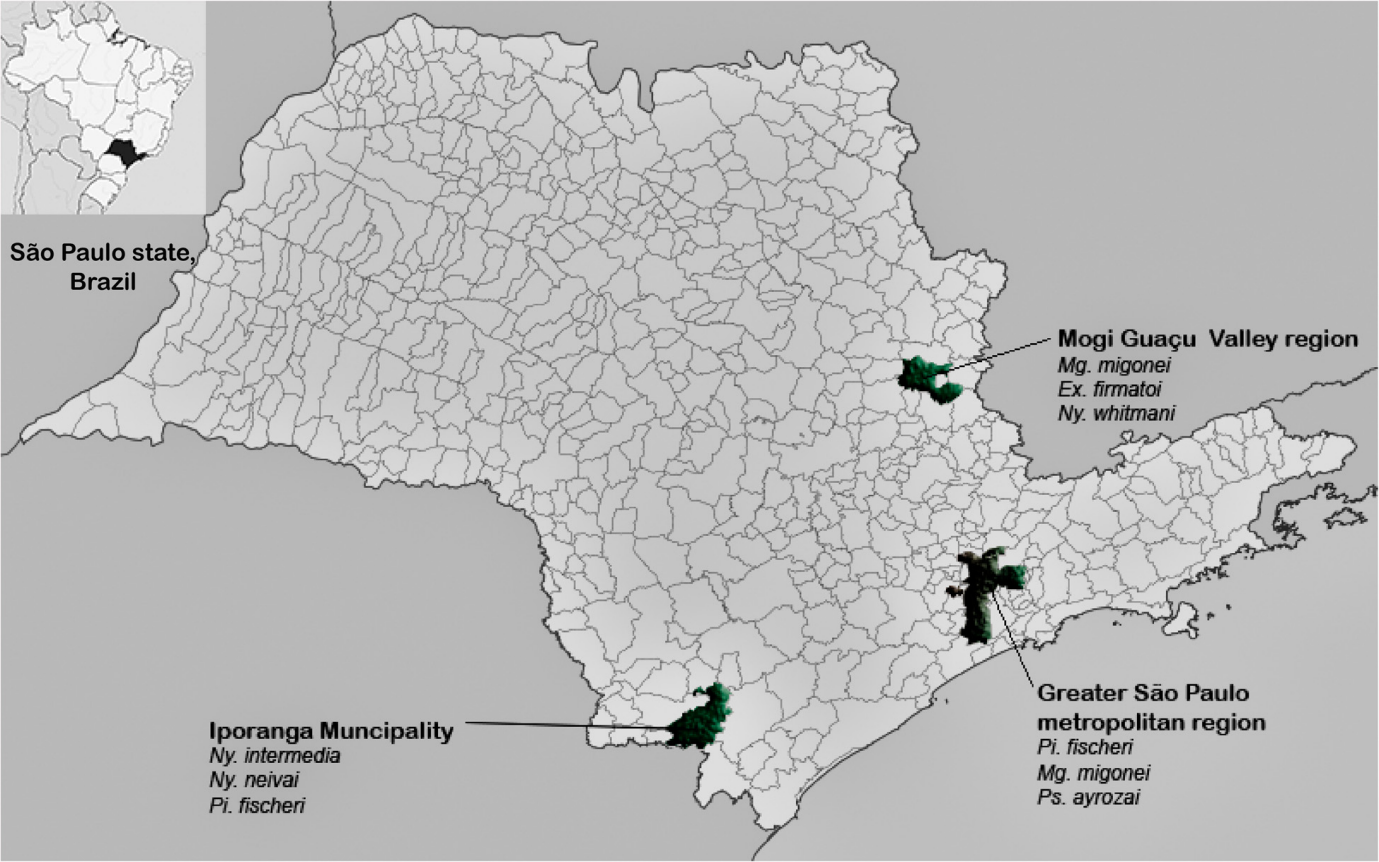
Localities where the sand flies species were captured in São Paulo state (SP), Brazil.

The species analyzed were captured in São Paulo state (SP), Brazil, in forest or peridomiciles close to the forest edge, in several localities within three regions (Figure 1):

*Mogi Guaçu Valley region* - northeastern SP - On a smallholding in the municipality of Mogi-Guaçu (22°20.041′ S, 46°50.755′ W; 671 m a.s.l. *Ex. firmatoi*, *Mg. migonei* and *Ny. whitmani* were collected at this site.

*Greater São Paulo metropolitan region*– eastern SP: at three sites: 1) on a smallholding (23° 37.656′ S, 46° 53.164′ W; 808 m. a.s.l) in Embu das Artes municipality; 2) on a smallholding (23°37′ 28″ S, 46°53′ 29″ W, 821 m a.s.l.) in Cotia municipality and 3) in the Parque Estadual da Cantareira (23°26′ 50.13″ S, 46°37′ 59.87″ W; .843 m. a.s.l.) São Paulo municipality. *Mg. migonei*, *Pi. fischeri* and *Ps. ayrozai* were collected in this region.

*Ribeira Valley region*- southeastern SP. On a small farm situated in the Serra district (24°33′ 19.7′ S; 48°40′42″ W; 198 m a.s.l.) in Iporanga municipality. *Mg. migonei, Ny. intermedia*, *Ny. neivai* and *Pi. fischeri* were collected at this site.

The collections were undertaken with modified black and white Shannon traps [26] installed from 18:00 to 23:00 hours and also with automatic modified CDC light traps, to which a small nylon cage (20 cm × 20 cm × 20 cm) was attached by means of a sleeve of *ca*. 40 cm in length, from 18:00 to 06:00 hours.

In the Shannon traps, the insects were captured with a Castro aspirator and the specimens were transferred to small nylon cages in which they were transported to the laboratory. The cages with the insects captured with CDC and Shannon traps were covered with damp towels to maintain the humidity. Hamsters were introduced into the cages in the Laboratorio de Entomologia em Saúde Pública/Phlebotominae of the Faculdade de Saúde Pública of the Universidade de São Paulo (LESP/FSP/USP) so that the females could feed and egg-laying be stimulated. The insects were maintained in a room at temperatures of 25 ± 1°C and humidity *ca.* 80%. The rearing of sandflies to obtain first- generation specimens was performed as described by [27].

Some first-generation specimens were used in the experiments with hamsters infected by *L.(V.) braziliensis* (strain MHOM/BR/1995/M15280), maintained in the Laboratório de Patologia de Moléstias Infecciosas of the Faculdade de Medicina of the Universidade de São Paulo (LIM-50-FMUSP). The experimental infections were undertaken in the LESP/FSP/USP.

The procedures for obtaining the parameters investigated were:

Proportion of females feeding on the host (hamster)

Females and males of each of the seven species were released into a nylon cage, where a non-infected or infected hamster under anesthetic was laid supine for about one hour, in the evening. The anesthetic was applied in accordance with the animal’s body weight: ketamine (15 mg/kg) + xilazine (0.05 mg/kg).In each experiment one hamster was exposed.

Duration of the gonotrophic cycle

The females after feeding on the hamsters were individually placed in a vial (2.5 cm in diameter and 7 cm in height) which was covered with a nylon cloth above which was a cover with a central hole through was introduced a small cotton swab moistened with sugar solution. The females were maintained in styrofoam boxes at a room temperature of 25° ± 1°C and humidity *ca*.80% and were observed twice daily, in the morning and early evening.The duration of the gonotrophic cycle was estimated by the median time (days) elapsed between blood feeding in hamsters and egg laying.

**Experimental infections of the sandfly species by *Leishmania braziliensis***

Males and females of each of the sand fly species were released together in a cage on the floor of which an infective anesthetized hamster (having a sore on each of its hind paws) was laid supine for about one hour in the evening. In each infection experiment one hamster was exposed. In the majority of experiments, the hamsters had their bodies covered with a nylon cloth, so that the female insects might bite only the hind paws with the wound. However, in one experiment with *Pi. fischeri* two hamsters were exposed simultaneously in two different cages, one with uncovered body, and the other only with exposed hind legs. The female dissection to expose the gut (to observe flagellates) and spermathecae (to identify the sandfly species) was carried out on a slide containing a drop of saline under a stereoscope (magnitude 60×). A cover glass covered the gut and genitalia which were examined under the microscope (magnification 400×). For a better view of the spermathecae and the flagellates in the gut a slight pressure was applied to the coverslip with the tip of a forceps. The dissection of the females which died naturally during the night was undertaken at 08:00 and of those which died during the day at 20:00 hours, approximately. The rate of infection of each sand fly species was calculated by the number of females with flagellates in their anterior, median and/or posterior gut observed under the microscope, divided by the total of engorged females. The identification of metacyclic promastigotes (late-stage infection forms) was undertaken considering the morphology of the flagellates: elongated body presenting flagellum three or more times longer than the body [28,29]. The frequencies of potentially infective females were obtained by the number of females with at least one infective form of the promastigotes observed in their anterior or thoracic gut divided by the total number of infected females.

Extrinsic incubation period

This parameter was obtained using the same methodology described for the infection rate. The extrinsic incubation period was calculated as the median duration (in days) between blood feeding and the presence of metacyclic forms in the anterior (head and esophagus) or thoracic gut (immediately after the estomodeal valve) [28,29].

This study was approved by the Ethics Committee on Animal Research of the Medical School of São Paulo University (CEP-IMT 057/2009) and the Biosafety Committee of the Faculty of Public Health, University of São Paulo (protocol 2026 OF.COEP/14/10).

## Results

### Proportion of females feeding on hamster

The numbers of experiments and of females, by species and collection locality, to which the hamsters (non-infected or infected) were exposed and the proportion of engorged females are given in Table 1.

**Table 1:** **Sand fly females by speciesand locality fed on hamsters both infected and uninfected by *Leishmania (Viannia) braziliensis *** **and numbers of experiments**

	**Exposed hamster’s condition**	**Uninfected**				**Infected, with only ulcerated legs exposed**			
**Species**	**Localities**	**Numbers of females exposed**	**Numbers of females engorged**	**Median proportions of engorged females**	**Numbers of experiments**	**Numbers of females exposed**	**Numbers of females engorged**	**Median proportions of engorged females**	**Numbers of experiments**
*Ex. firmatoi*	Mogi Guacu	141	63	0.42	4	21	4	0.19	1
*Mg. migonei*	Embu das Artes	28	6	0	5	12	4	0.20	5
	Lab (M.Guaçu)	…	…	…	…	8	7	0.88	1
	Iporanga	…	…	…	…	21	8	0.38	2
	Mogi Guaçu	99	65	0.60	4	32	7	0.22	1
*Ny. intermedia*	Iporanga	…	…	…	…	852	311	0.32	6
*Ny. neivai*	Iporanga	…	…	…	…	99	45	0.25	7
*Ny. whitmani*	Mogi Guaçu	1164	384	0.33	3	93	22	0.31	1
*Pi. fischeri*	Cotia	269	128	0.59	9	…	…	…	…
	Embu das Artes	449	202	0.46	14	347	151	0.22	5
	Cantareira	408	310	0.77	2	203	93	0.46	2
	Iporanga	…	…	…	…	132	61	0.32	4
*Ps. ayrozai*	Cantareira	19	0	0.00	4	232	5	0.02	1

…no experiment undertaken; Lab (M. Guaçu) = F1 obtained in laboratory from females captured in Mogi Guaçu municipality.

In the experiments with non-infected hamster, *Pi. fischeri* was the species which underwent the greatest number of experiments (25) and provided the greatest number of exposed females (1,844) from the three sites in the Greater São Paulo region; the median of the proportions of engorged females varying between 0.46 and 0.77. For *Mg. migonei*, a total of 127 females were exposed in nine experiments with specimens from Embu das Artes and Mogi Guaçu; the median of the proportion of females engorged varying between nil and 0.6. Regarding the two other species from Mogi Guaçu, three experiments were undertaken with *Ny. whitmani* involving 1,164 exposed females; the median value of the proportion of females engorged being 0.33. As for *Ex. firmatoi*, four experiments were carried out with a total of 141 females; the median value of engorged females being 0.42. Four experiments were undertaken involving 19 females of *Ps. ayrozai*, none of them engorged (Table 1).

Regarding the experiments involving hamsters with only the ulcerated paws exposed infected by *L. (V.) braziliensis*, for *Pi. fischeri,* 11 experiments were carried out, seven with females from Greater São Paulo (Embu das Artes and Cantareira) and four from Ribeira Valley (Iporanga), with a total of 305 engorged females out of a total of 682 exposed (0.45); the median values of the proportion varying between 0.22 and 0.46. Regarding *Mg. migonei*, a total of 26 engorged females of 73 exposed (0.36) was observed in a total of nine experiments with specimens from the three regions; the median proportion of females engorged varying between 0.20 and 0.88, the highest proportion of them being from one of the experiments with F1 females raised in the laboratory. As for the two species from the Ribeira Valley, *Ny. intermedia* and *Ny. neivai*, six and seven experiments were, respectively, undertaken with each. For *Ny. intermedia* 852 females were exposed while for *Ny. neivai*, 99; the median values of engorged females being respectively 0.32 and 0.25. For *Ny. whitmani*, only one experiment was carried out with 93 females exposed and with a proportion of engorged females of 0.31.The lowest proportion of engorged females 5/232 (0.02) was observed for *Ps. ayrozai* (Table 1).

In the single experiment undertaken with *Pi. fischeri* blood feeding on a hamster infected with its body uncovered, 172 females were exposed and 133 engorged (0.77).

### Gonotrophic cycle

The gonotrophic cycles of *Ex. firmatoi*, *Mg. migonei*, *Ny. intermedia*, *Ny. neivai*, *Ny. whitmani* and *Pi. fischeri* are given in Table 2. This period varied from 4 to 8 days; the median for *Mg. migonei* was 7 days, for *Ny. neivai* and *Ny. whitmani* 4 days and for the other three species 5 days.

**Table 2:** **Gonotrophic cycles of *Ex. firmatoi*, *Ny. intermedia*, *Ny. neivai*, *Ny. whitmani *** **and *Pi. fischeri *** **under laboratory conditions**

**Species**	**N**	**Minimum (days)**	**Maximum (days)**	**Median (days)**
*Ex. firmatoi*	46	3	7	5
*Ny. intermedia*	48	4	7	5
*Ny. neivai*	44	3	6	4
*Ny. whitmani*	62	3	7	4
*Mg. migonei*	50	7	8	7
*Pi. fischeri*	150	4	8	5

### Infection and late-stage infection rates

The infection rates of *Ex. firmatoi*, *Mg. migonei*, *Ny. intermedia*, *Ny. neivai, Ny. whitmani* and *Pi. fischeri* by *L. braziliensis* and their extrinsic incubation periods in these six sand fly species which blood fed on hamsters infected with only the ulcerated paws exposed are presented in Table 3. A total of 635 females representing the six sand fly species were observed, *Ex. firmatoi* (4.3%), *Mg. mig*onei (3.9%), *Ny. intermedia* (40.5%), *Ny. neivai* (7.1%), *Ny. whitmani* (9.6%) and *Pi. fischeri* (34.6%). The highest infection rate was observed for *Ny. whitmani* (34.4%) and the lowest for *Ny. neivai* (13.3%) and *Ny. intermedia* (6.2%). *Ex. firmatoi*, *Mg. migonei* and *Pi. fischeri* presented intermediate values between 22.2% - 24.0%.

**Table 3:** **Numbers of females by sandfly species, according to the number of days elapsed between their feeding and dissection, and the infected and potentially infective condition by *Leishmania (Viannia) braziliensis***

**Species**	***Ex. firmatoi***		***Mg. migonei***		***Ny. intermedia***		***Ny. neivai***		***Ny. whitmani***		***Pi. fischeri***	
**Day after feeding**	**dis**	**inf (pif)**	**dis**	**inf (pif)**	**dis**	**inf (pif)**	**dis**	**inf (pif)**	**dis**	**inf (pif)**	**dis**	**inf (pif)**
1st	…	…	1	0	4	0	…	…	…	…	7	0
2nd	…	…	3	0	28	0	…	…	6	0	19	0
3rd	3	1 (0)	0	0	10	0	1	0	…	…	17	4 (0)
4th	9	1 (1)	7	1	75	4 (4)	8	1 (1)	16	5 (5)	49	12 (3)
5th	14	4 (4)	14	5 (3)	122	8 (8)	30	3 (3)	35	16 (16)	58	19 (17)
6th	1	0	…	…	13	2 (2)	5	1 (1)	4	0	36	11 (11)
7th	…	…	…	…	5	2 (2)	1	1 (1)	…	…	34	5 (5)
**Total**	27	6 (5)	25	6 (3)	257	16 (16)	45	6 (5)	61	21 (21)	220	51 (36)
Infected (%)	22.2	24.0	6.2	13.3	34.4	23.2						
Potentially Infective/infected (%)	**83.3**		**50.0**		**100.0**		**83.3**		**100.0**		**70.6**	
Surviving % after egg- laying	**3.7**		**0**		**7.0**		**80.0**		**65.6**		**31.8**	

dis = dissected females; inf = infected females; (pif) = potentially infective females; … not observed.

* = median of gonotrophic cycle period.

For *Ps. ayrozai*, no infected female was observed among the five which engorged on an infected hamster.

No females of *Mg. migonei* survived beyond the 5th day after the blood meal, nor those of *Ex. firmatoi* and *Ny. whitmani* after the 6th day or those of *Ny. intermedia*, *Ny. neivai* and *Pi. fischeri* beyond the 7th day.

For *Ny. intermedia* and *Ny. whitmani*, 100% of the females infected with flagellates presented late-stage infection forms, while only 50% did so in *Mg. migonei;* intermediate values (70.6% - 83.3%) being observed for *Pi. fischeri*, *Ny. neivai* and *Ex. firmatoi*.

As regards the females of *Pi. fischeri* exposed to hamsters infected by *L. (V.) braziliensis* with its whole body exposed - of a total of 109 females dissected only 6 (5.5%) developed infection and in 83.3% of these (5/6) the forms were potentially infective. In this experiment, 14/30 (47%) females dissected presented blood in the gut until the 4^th^ day after feeding. No further remnants of blood were observed in the gut of the females dissected after the 5^th^ day (Table 4).

**Table 4:** **Numbers of females of *Pintomyia fischeri *** **dissected by post-blood meal on a hamster infected by *Leishmania braziliensis *** **with the whole body exposed, presence of blood in the gut, infected by the parasite and presence of late-stage of development promastigotes**

**Day of the blood meal**	**Dissected**	**With blood in the gut**	**Infected**	**Late-stage development promastigotes**
2nd	2	2	0	0
3rd	7	7	0	0
4th	30	14	1	0
5th	3	0	0	0
6th	39	0	3	3
7th	21	0	1	1
8th	7	0	1	1
Total	109	23	6	5
%		12.8	5.5	4.6

### Extrinsic incubation period

*Migonemyia migonei* presented potentially infective forms on the 5th day after feeding, but the other species’ potentially infective forms were observed as from the 4^th^ day (Table 2). However, for *Pi. fischeri* feeding on infected hamsters with the body uncovered the extrinsic incubation period occurred as from the 6th day; but only three females died on the 5th day.

## Discussion

### Blood feeding habit

For the female insects which present the gonotrophic concordance (each blood meal is followed by one oviposition) frequently observed among sand fly species [30], the vector’s biting habit could be estimated indirectly by the proportion of insects which feed on the source host or one susceptible to infection by a particular agent, divided by the period (in days) of the gonotrophic cycle [24]. This value being a quotient of these two measurements, for populations with disharmonic gonotrophic concordance (each egg-laying is dependent on two or more blood meals – as, e.g., in the case of *Phlebotomus papatasi*) [31] the biting habit only contributes to raising the vectorial capacity if the period of extrinsic incubation is completed before the next meal. Populations which present this characteristic have greater epidemiological significance as potential vectors. However, the period of extrinsic incubation has been shown to be very close to that of the gonotrophic cycle. This latter, in sand flies which feed on warm-blooded animals, varies from 4 to 10 days, frequently from the seventh to the tenth day [30].

In this study the median duration of the gonotrophic cycle for the species studied was between the fourth and fifth day (Table 4), however, this parameter may vary depending on the temperature. For *Ny. neivai* under natural conditions it was estimated at 8 days [32].

In natural circumstances, apart from the preference of hematophagous insects for feeding on particular hosts, the biting rate is influenced by the diversity, density and spatial and temporal distribution of the blood source populations [33-35].

Although in this present study one single type of host (a rodent) was exposed, it was observed that of the seven sand fly species investigated, *Ps. ayrozai* showed the lowest biting rate, suggesting the low attractiveness of this animal for this species. This low attractiveness was evidenced because no *Ps. ayrozai* female bit the non-infected hamster with its body uncovered and only 2% of the females bit the infected paws when they were exposed (Table 1). Similarly, the low attractiveness of another rodent (a guinea-pig) to this species was observed in natural conditions (0.57 insect/night/bait) when compared to that of the armadillo (*Dasypus novemcinctus*) (73.7/insects/night/bait) in Montagne des Chevaux, French Guyana [36].

*Psychodopygus ayrozai* is one of the most frequent species in primary Atlantic forest, being collected both at ground level and in the forest canopy, and has been shown to be anthropophilic [20,37-40]. Despite these characteristics of this sand fly, the transmission of *Leishmania* to the human population in primary Atlantic forests is low and has been associated with the zoophilic habits of the vectors [39]. However, if the weak hamster attractiveness to *Ps. ayrozai* observed in the laboratory experiments can be extrapolated to wild rodents (significant reservoirs of *Leishmania braziliensis*) [41], the low transmission of this *Leishmania* in this biome could also be explained as due to the preference of this sand fly for feeding on animals which play little or no role as reservoirs of the cutaneous leishmaniasis agents (e.g. armadillos) in this environment.

In the present study, for the sand fly species captured in the same localities, challenged to bite the hamsters with only the infected paws or with the whole body uncovered: *Pi. fischeri* from Embu das Artes and Cantareira, *Ex. firmatoi* from Mogi Guaçu and *Mg. migonei* from Embu das Artes and Mogi Guaçu, it was observed that the proportion of engorged females was also greater on the uncovered hamster than on that which had only its paws exposed (Table 1). This may be explained by the larger exposed area and consequently lower competitiveness for space for landing to bite. However, the infection rate was greater when only the paws with sores were exposed. This seems to show that the *Leishmania braziliensis* is concentrated in the sore; even so the infection rate was greater than that observed for experimental infection by *Leishmania tropica* in *Phlebotornus duboscqi* females fed on hamster lesions [42]. A low proportion of those engorged was observed when few females were used in the experiments. This fact may be associated with the liberation of pheromones of aggregation which stimulate the landing on the host and the haematophagy [43,44].

Regarding *Ny. neivai,* this species was considered a junior synonym of *Ny. intermedia* for nearly 70 years [45]. Thus, despite there being some information in the literature about the species’ attractiveness and the related experimental infection it probably refers, in fact, to *Ny. intermedia s. lat.* in the Southeast region of Brazil, *Ny. intermedia* and *Ny. neivai* are anthropophilic species and are captured in high frequencies in the intra and peridomicile. *Nyssomyia intermedia* is associated with the coastal region but in São Paulo state *Ny. neivai* occurs exclusively in the plateau region and sympatrically with *Ny. intermedia* in the Ribeira Valley region [20,46]. The anthropophily of *Ny. intermedias. lat.* in a forested environment in the Ribeira Valley region (sympatric area of *Ny. neivai* and *Ny. intermedia*) was estimated at 9.3/females/night [47] and for *Ny. intermedia* populations in Espírito Santo state in an area far from the coast, an average of 6.7 insects/night was observed [48]. However, in a forested coastal area, the attractiveness of human for this species both in São Paulo and Espírito Santo states was greatly reduced [16,48]. For these two species of this sympatric area the proportions of engorged females were very close (Table 1).

### Infection rate and infective potential

In the experiments involving the six sand fly species with positive results for *L. (V.) braziliensis* infection, the median rate of infected insects varied from 6.2 to 34.4%; the lowest of them being for *Ny. intermedia* and the highest for *Ny. whitmani*. The median value of 6.2% found for wild females of *Ny. intermedia* was much lower than the infection rates observed for females of the laboratory colony (39^th^ generation) whose forebears came from Rio de Janeiro state [49,50]. They observed an infection rate above 30% for females fed on an infected hamster or artificially on chicken-skin membrane. This disparity may be related to the population differences between sand fly species, parasite load or size and time of exposure of the ulcered hamster. However, this median value was greater than that observed in experiments with laboratory females coming from the same area as those of the present study and fed on a hamster infected by *Leishmania (Leishmania) amazonensis* 1/26 (3.8%) [51].

*Nyssomyia neivai* presented a median value 2.1 times greater than that of *Ny. intermedia* (Table 2). However, both species presented much lower values than those for *Ny. intermedia s. lat*. whether obtained in the laboratory (F1) or in the wild (varying from 67% to 76%), also coming from the Ribeira Valley region [52]. Although the proportion of potentially infective females was lower for *Ny. neivai* (83.3%) than for *Ny. intermedia* (100.0%), our result suggests that the *Ny. neivai* population presents a higher probability of infection and infectivity by *L. (V.) braziliensis* than *Ny. intermedia*. This because the exposition of the infected hamster to these two sand fly species collected together took place simultaneously.

Despite the sample of *Ny. neivai* having been considerably inferior in number to that of *Ny. intermedia*, the mortality in this period was greater for *Ny. intermedia* (16%) than for *Ny. neivai* (2.2%). Considering the infectivity and the proportion of engorged females surviving after the complete extrinsic incubation period for *L. braziliensis,* our results suggest that *Ny. neivai* presents an infective survival rate greater than *Ny. intermedia*, having, therefore, a greater probability of taking a second infective meal on a susceptible host. Although wild females of unknown physiological state were used in the experiments, which might imply different mortality rates, a study of *Ny. intermedia s. lat.* in the Ribeira Valley, where the two species are sympatric, indicated that of 26,383 females captured, 99.4% of them were nulliparous, which suggests that there was a high proportion of young females among them [53]. Therefore, in the light of this information, a great proportion of young specimens among the females used in the experiments could be expected and so the age factor had little effect on the mortality rate.

Previous studies in the Ribeira Valley region showed that the ratio between *Ny. intermedia* and *Ny. neivai* in a peridomicile 180 m from forest was 3.5/1.0, while that in a peridomicile of a more anthropic area 700 m from the forest was 1.5/1.0 [20]. This information suggests that *Ny. neivai* is more adapted to the anthropic environment than is *Ny. intermedia*. Thus, if both species present the same degree of anthropophily in areas of great human influence, *Ny. neivai* presents a transmission potential of *Leishmania* sp. slightly higher than that of *Ny. intermedia,* while in a more preserved area that of *Ny. intermedia* would be double that of *Ny. neivai*.

As regards *Ny. whitmani*, a recognized vector of *L. (V.) braziliensis*[54], the values here found confirm its susceptibility to infection and the development of potentially infective forms (100%); it was, further, found to have the highest infection rates. An experimental infection rate for this sand fly species of 2% (1/46) was observed when it was fed on an ulcer on an infected monkey of the *Rhesus* genus [14]. On the other hand, specimens of this sand fly species from Bahia feeding on an ulcer on a dog present rates varying from 1.8 to 8.3% [38]. The data here presented for *Ny. intermedia* and *Ny. whitmani* show that the infection rate of this latter is 5.3 times greater. However, for both species a similar proportion (100.0%) of the females harboring late-stage infection forms among those infected was observed (Table 1). This, together with the greater degree of attachment of the procyclic forms to the gut epithelium in *Ny. whitmani* than in *Ny. intermedia* (~10 folds) [55] seems to explain our results, since a lower level of gut attachment of the procyclics reduces the chances of success of their later phases. Once this barrier is overcome, the parasites in both species would have similar chances of attaining the metacyclic stage. However, distinct parasite loads among hamsters exposed to the bite of these two species cannot be ignored when we seek to explain their different infective rates.

*Pintomyia fischeri* is a common species in foci of cutaneous leishmaniasis in the Southeast, South and Northeast regions of Brazil [10-13]. Although it is more frequently found in forests, it also occurs in anthropic environments [11-13]. Despite its having been considered a possible vector of *L. (V.) braziliensis* on the basis of its high degree of anthropophily, frequency in the intradomicile and susceptibility to experimental infections when fed on monkeys of the *Rhesus* genus (0.4%; 1/246) [12], only recently has its natural infection by *L. braziliensis* or by *L. (Viannia*) been demonstrated [15,56-58]. The infection rate of 23.2% observed for *Pi. fischeri* in the present study reinforces the hypothesis of its participation in the transmission of this parasite, mainly in the Greater São Paulo area, where it predominates over the other species [12,13,59] and is anthropophilic [12,13]. *Pi. fischeri* also predominated in captures on human bait in a residual forest close to domiciles situated in the central-northeast region (in an area of Paleozoic depression, 600-800 m a.s.l.) of São Paulo state and also in Espírito Santo state in forest close to the coast, but in this latter area in an anthropic environment its frequency was less than that of *Ny. intermedia,* and in a municipality (Afonso Claudio) further inland in Espirito Santo state its frequency was inferior to that of other vectors [48].

*Migonemyia migonei,* an inhabitant of primary and secondary forests, which also frequents human dwellings, feeding avidly on humans and domestic animals (dogs and birds), contributed 14% of the sand fly specimens collected in São Paulo state in the early 1940s [12]. Its natural infection rate possibly by *L. (V.) braziliensis* (0.2%; 6/2842) was observed in the northwest region of São Paulo state, a highly endemic area [14] and currently this species is considered a secondary vector of the cutaneous leishmaniasis agent in this state [10]. In Baturité municipality in Ceará state it was the third most frequent species and was found to be naturally infected with *L. (V.) braziliensis*[60,61]; in two foci in Rio de Janeiro municipality it was the second most prevalent species and found naturally infected with *L. (V.) braziliensis*[62] and in São Vicente Férrer in Pernambuco state it was the most prevalent species in a domiciliary environment and found naturally infected [63]. In the present study an infection rate of 24.0% was obtained - lower than the approximately 50% observed in sand flies of this species fed artificially [64]. So in the light of various factors: the infection and potentially infective rates observed in the present study, the rodents’ attractiveness to this species under natural conditions [48] and several findings of specimens with natural infection by *L. (V.) braziliensis* or *Leishmania* (*Viannia*) sp, this sand fly may be seen as in fact playing an important role in the transmission of cutaneous leishmaniasis agents.

*Expapillata firmatoi* presented an infection rate of 22.2%. However, despite its anthropophilic habit of biting in both diurnal and nocturnal periods and its being one of the most frequent species in the northeastern region of São Paulo state [39], this species has not been found naturally infected by *Leishmania*. However, in forest in Espírito Santo state (Afonso Claudio municipality) it was little attracted to human bait, but was the species most attracted to Disney traps baited with the primates *Cebus apela* and *Callithrix geoffroy*, marsupials (*Marmosa cinerea*, *Philander opossum* and *Metachirus nudicaudatus*) and the rodent *Agouti paca*[48]. Thus its role as a potential vector of cutaneous leishmaniasis agents still calls for elucidation.

Although the dissection technique used in this present study could lead to the underestimation of infection rates for the sand fly species observed, as can be inferred from some studies that beyond this technique also used molecular tests [63-67] and also because the females were dissected as they were dying (thus increasing the chances of degradation of the parasite with negative results under microscopic examination), it was possible on the other hand to observe the potentially infective promastigote in the thoracic mid-gut of the sand fly and the extrinsic incubation period compatible with the transmission of the parasite during the next blood meal. This information together with the data related to high frequencies, anthropophily, presence in domiciliary environment and the findings of natural infection by *L. (V.) braziliensis* or *L*. (*Viannia*) sp. of the sand fly species investigated, strengthens the evidence incriminating them as vectors of *L. braziliensis,* whether acting in isolation or in sympatry in foci scattered over a large part of Brazil, Argentina and Paraguay.

## Conclusions

Of the seven species investigated, *Ex. firmatoi, Mg. migonei, Ny. intermedia*, *Ny. neivai*, *Ny. whitmani*, *Pi. fischeri* and *Ps. ayrozai*, the first six presented a relatively high proportion feeding on hamsters and shown to be susceptible to experimental infection by *L. (V.) braziliensis*. These also presented promastigotes in late-stage of development in their thoracic mid-gut and the extrinsic incubation period compatible with the gonotrophic cycle. Our results together with other parameters of their behavior under natural conditions, suggest the potential role of *Ex. firmatoi* as vector of this parasite and reinforce that of *Mg. migonei, Pi. fischeri, Ny. neivai, Ny. intermedia* and *Ny. whitmani* in the areas in which they occur.

## Competing interests

The authors declare that they have no competing interests.

## Authors’ contributions

EABG, FGO designed and supervised the study. MMCSD, FGO, EABG, CCG, COL undertook field and laboratory activities. EABG, FGO, analyzed the data, drafted and revised the manuscript. All the authors revised and approved the final version of the manuscript.
